# Supplementary material for: Flotillin-associated rhodopsin (FArhodopsin), a widespread paralog of proteorhodopsin in aquatic bacteria with streamlined genomes
Source: mSystems. 2023 May 24;8(3):e00008-23. doi: 10.1128/msystems.00008-23 (PMC10308929; doi:10.1128/msystems.00008-23)
Supplement: FIG S1 — Complete protein sequence alignment of six proteorhodopsin and six FArhodopsin sequences. Sequences come from six SAGs (two of each taxa) containing both rhodopsins in their genomes. Dark and light grey regions represent conserved residues within the alignment. [file msystems.00008-23-s0001.pdf]

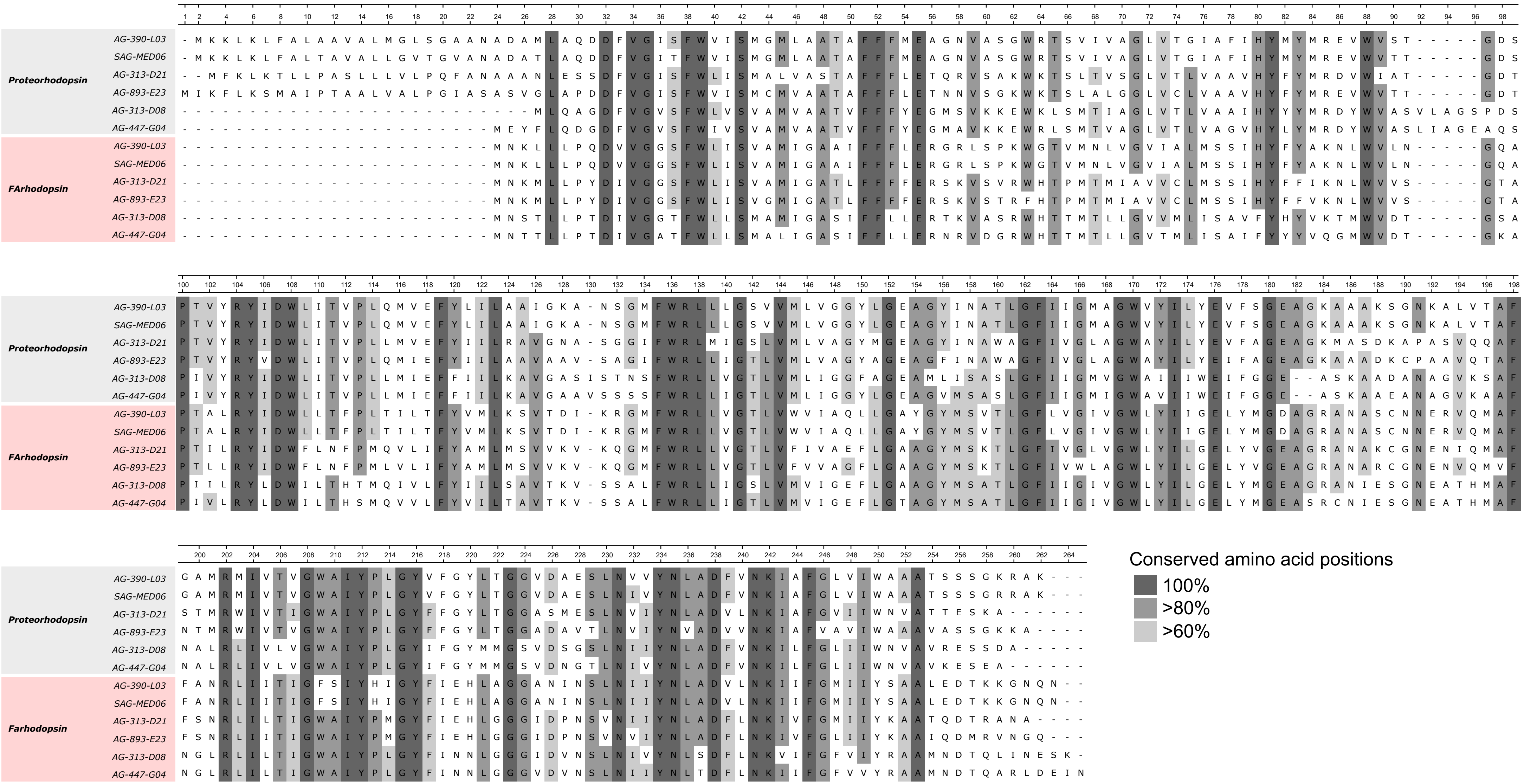

Proteorhodopsin
AG-390-L03
SAG-MED06
AG-313-D21
AG-893-E23
AG-313-D08
AG-447-G04
FArhodopsin
AG-390-L03
SAG-MED06
AG-313-D21
AG-893-E23
AG-313-D08
AG-447-G04
Farhodopsin
Conserved amino acid positions
100%
>80%
>60%
