# Supplementary figures and images for: Flotillin-associated rhodopsin (FArhodopsin), a widespread paralog of proteorhodopsin in aquatic bacteria with streamlined genomes
Source: mSystems. 2023 May 24;8(3):e00008-23. doi: 10.1128/msystems.00008-23 (PMC10308929; doi:10.1128/msystems.00008-23)

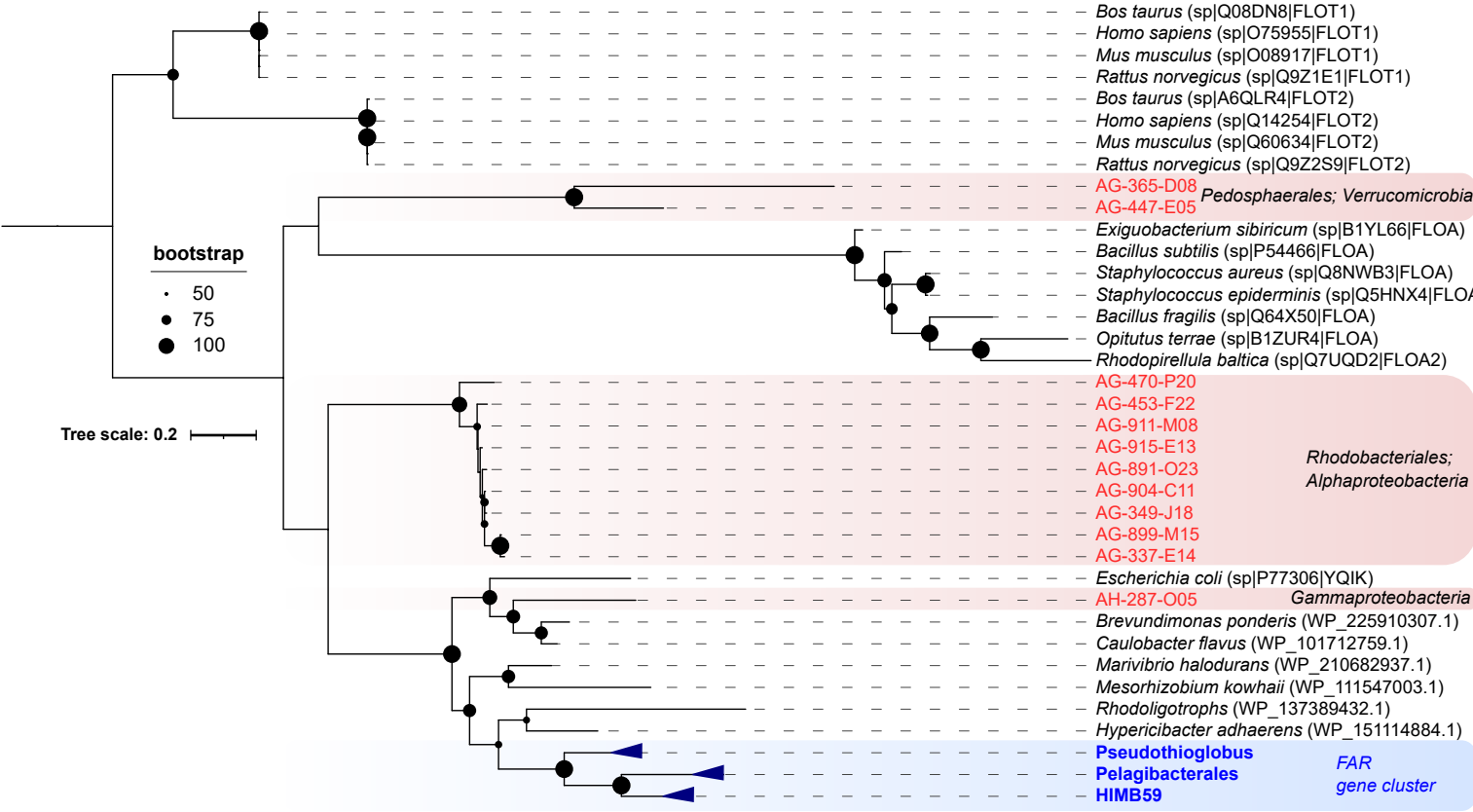

Supplement: FIG S2 — Maximum-likelihood phylogenetic tree of flotillin genes retrieved from marine SAGs. Reference flotillin sequences and their accession numbers within brackets are colored in black. Sequences in red represent sequences from marine SAGs that were not found near a FArhodopsin. Sequences in blue correspond to the flotillins of the three taxa coding for the tandem flotillin-FArhodopsin. Sequences in this branch were condensed for simplicity. [file msystems.00008-23-s0002.pdf]

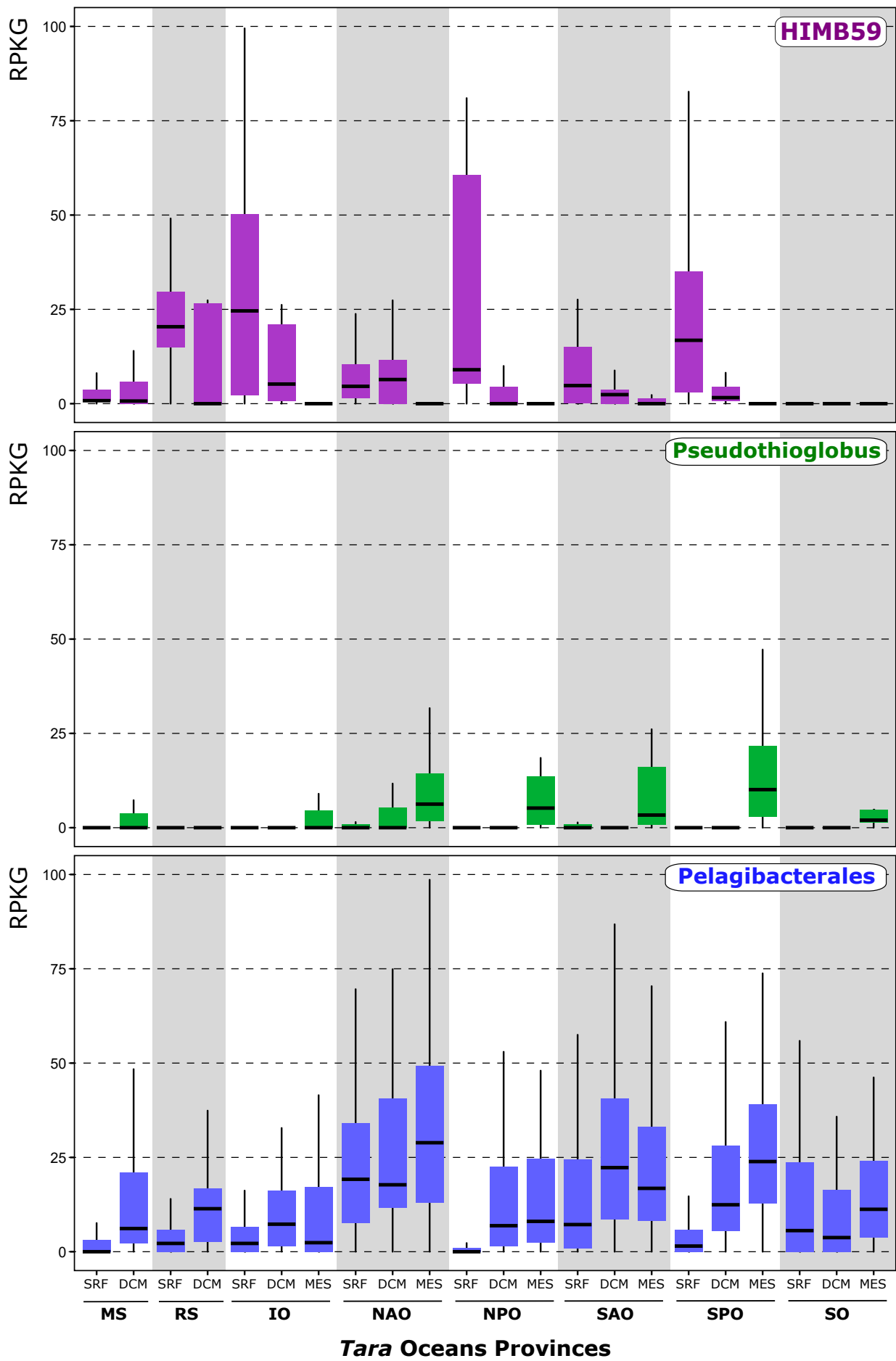

Supplement: FIG S3 — Relative abundance (measured in RPKG) of the FAR gene cluster in Tara Ocean metagenomes. Each province has been divided by depth in surface (SRF), deep chlorophyll maximum (DCM), and mesopelagic (MES) regions. The bars have been colored according to the microbes (blue, Pelagibacterales; purple, HIMB59; and green, Pseudothioglobus). [file msystems.00008-23-s0003.pdf]
